# Single-Ion Conducting Polymer Nanoparticles as Functional Fillers for Solid Electrolytes in Lithium Metal Batteries

**DOI:** 10.1021/acsami.1c15771

**Published:** 2021-11-03

**Authors:** Luca Porcarelli, Preston Sutton, Vera Bocharova, Robert H. Aguirresarobe, Haijin Zhu, Nicolas Goujon, Jose R. Leiza, Alexei Sokolov, Maria Forsyth, David Mecerreyes

**Affiliations:** † POLYMAT University of the Basque Country UPV/EHU, Joxe Mari Korta Center, Avenida Tolosa 72, 20018, Donostia−San Sebastian, Spain; ‡ ARC Centre of Excellence for Electromaterials Science and Institute for Frontier Materials, Deakin University, Melbourne, 3125 Australia; § Chemical Sciences Division, Oak Ridge National Laboratory, Oak Ridge, Tennessee 37831, United States; ∥ Department of Chemistry, University of Tennessee, Knoxville, Tennessee 37996, United States; ⊥ Ikerbasque, Basque Foundation for Science, Maria Diaz de Haro 3, E−48011 Bilbao, Spain

**Keywords:** single-ion, nanoparticle, lithium, electrolyte, gel, solid-state, battery

## Abstract

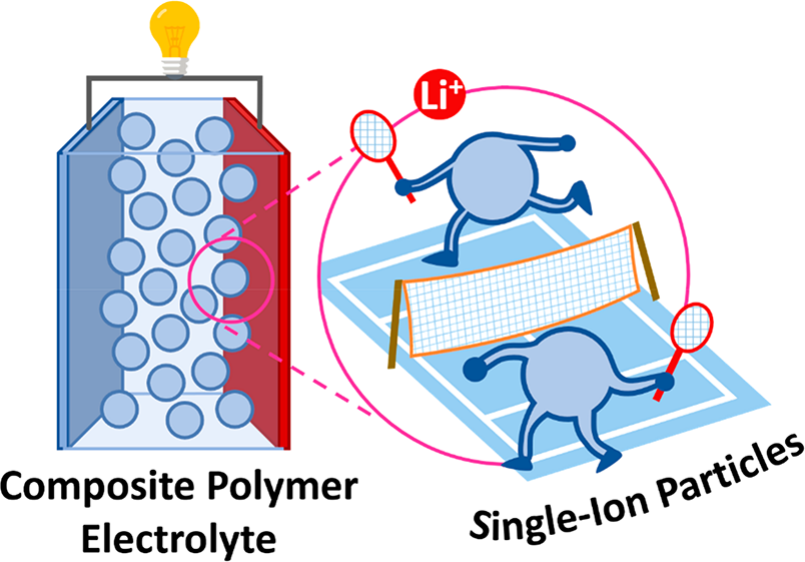

Composite solid electrolytes including inorganic nanoparticles or nanofibers which improve the performance of polymer electrolytes due to their superior mechanical, ionic conductivity, or lithium transference number are actively being researched for applications in lithium metal batteries. However, inorganic nanoparticles present limitations such as tedious surface functionalization and agglomeration issues and poor homogeneity at high concentrations in polymer matrixes. In this work, we report on polymer nanoparticles with a lithium sulfonamide surface functionality (LiPNP) for application as electrolytes in lithium metal batteries. The particles are prepared by semibatch emulsion polymerization, an easily up-scalable technique. LiPNPs are used to prepare two different families of particle-reinforced solid electrolytes. When mixed with poly(ethylene oxide) and lithium bis(trifluoromethane)sulfonimide (LiTFSI/PEO), the particles invoke a significant stiffening effect (*E*′ > 10^6^ Pa vs 10^5^ Pa at 80 °C) while the membranes retain high ionic conductivity (σ = 6.6 × 10^–4^ S cm^–1^). Preliminary testing in LiFePO_4_ lithium metal cells showed promising performance of the PEO nanocomposite electrolytes. By mixing the particles with propylene carbonate without any additional salt, we obtain true single-ion conducting gel electrolytes, as the lithium sulfonamide surface functionalities are the only sources of lithium ions in the system. The gel electrolytes are mechanically robust (up to *G*′ = 10^6^ Pa) and show ionic conductivity up to 10^–4^ S cm^–1^. Finally, the PC nanocomposite electrolytes were tested in symmetrical lithium cells. Our findings suggest that all-polymer nanoparticles could represent a new building block material for solid-state lithium metal battery applications.

## 1 Introduction

Solid-state lithium batteries (SSLBs) have the potential to extend the range of electric vehicles and enable large-scale storage for renewable energy. As the name implies, SSLBs have no liquid components, distinguishing them from traditional LIBs and resulting in safer and more energy-dense devices. The increased energy density of SSLBs is ascribed to a more compact design and the use of high capacity lithium metal electrodes, both of which hinge on the development of novel electrolyte materials.^1−3^ Solid electrolytes that show fast lithium conduction, while avoiding the common drawbacks of liquid electrolytes such as flammability and leakage, have been extensively studied in recent years for this purpose. Among the different types of solid electrolytes, composite electrolytes that combine several materials such as polymer matrixes, inorganic nanoparticles, nanofibers, organic solvents, ionic liquids, and salts are an emerging class.^4^ This is due to the limitations commonly typically shown by polymer (low ionic conductivity and lithium transference number) or inorganic solid electrolytes (low mechanical properties and interfacial stability).

An example of a composite system is obtained by mixing a conventional electrolyte matrix with nanosized fillers such as nanofibers or nanoparticles. Previous research on composite electrolytes of this type has focused on inorganic nanoparticles and polymeric nanofibers.^5^ For example, inorganic nanoparticles have been used to immobilize or thicken liquid electrolytes, i.e., organic carbonates, glymes, or ionic liquids, while polymeric nanofibers such as cellulose nanofibers or electrospun PVDF nanofibers^6,7^ have been used to mechanically reinforce electrolyte matrixes.^8,9^ Inorganic nanoparticles have also been dispersed into polymer electrolytes, such as poly(ethylene oxide), with the aim to increase mechanical properties and ionic conductivity due to the nanostructuration effect.^10−12^ However, inorganic nanoparticles suffer from agglomeration issues and poor homogeneity at high concentrations. Modifications of the inorganic nanoparticle surfaces with a polymeric shell or ionic functional groups were shown to improve nanoparticle dispersion and reduce particle agglomeration. Thus, functionalizing the nanoparticle surface with immobilized anions or polyanions has proven an effective strategy to prepare single-ion conducting electrolytes.^13−16^ Despite these considerable research efforts, the synthesis of functionalized inorganic nanoparticles on a large scale presents some applicability issues such as specific surface chemistries for the grafting process and tedious purification procedures.

Surprisingly, much less attention has been paid to the use of functionalized polymeric nanoparticles despite polymers offering a wide range of easily up-scalable techniques.^17−21^ For instance, emulsion polymerization, the more versatile polymerization technique to produce waterborne polymer dispersions, offers a straightforward and industrially feasible method to obtain polymer nanoparticles of a variety of compositions and with controllable and monodispersed sizes between 50 and 500 nm.^22,23^ Recently, Kim et al.^24^ reported the synthesis of ionic functional polymer nanoparticles by postfunctionalization strategies of polymer particles which, as indicated before, is the most straightforward option for mass production. Indeed, surface functionalization of the nanoparticles with ionic groups could be in principle carried out simply by including ionic monomers or polymerizable surfactants in the reaction formulation without the need for additional steps.

The first goal of this work is to investigate the synthesis of poly(methyl methacrylate)-functionalized polymer nanoparticles by a one-step and scalable emulsion polymerization process. The surface functionalization of the polymer nanoparticles will be attempted by including in the formulation the comonomer lithium 1-(3-(methacryloyloxy)propylsulfonyl)-1-(trifluoromethylsulfonyl)imide (LiMTFSI) which is a well-known monomer for obtaining lithium single-ion conducting polymer electrolytes.^25−27^ The second goal of the work is to show the potential of these Li single-ion functional polymer nanoparticles in composite solid electrolytes for lithium batteries through two examples. In the first example, the particles were mixed with a typical solid polymer electrolyte based on lithium bis(trifluoromethanesufonyl)imide and poly(ethylene oxide). The polymer electrolyte showed high ionic conductivity and improved mechanical properties with respect to the nanoparticle-free polymer electrolyte. In the second example, the nanoparticles were combined with propylene carbonate (PC) to prepare single-ion composite gel electrolytes. The nanoparticle electrolytes showed promising ionic conductivity values and transference number close to unity. Finally, the two composite solid electrolytes were tested in symmetrical lithium cells and LiFePO_4_ lithium metal cells to investigate their potential in SSLBs.

## 2 Experimental Procedures

### Materials

Methyl methacrylate (MMA) and ethylene glycol dimethacrylate (EGDMA) were purchased from Sigma-Aldrich and vacuum distilled before use. Lithium 1-(3-(methacryloyloxy)propylsulfonyl)-1-(trifluoromethylsulfonyl)imide (LiMTFSI) was purchased from Specific Polymers and used as is. Ascorbic acid, *tert*-butyl hydroperoxide (70% solution in water, TBHP), and poly(ethylene oxide) (average MW 900 000 g/mol, PEO) were purchased from Sigma-Aldrich and used as is. Lithium bis(trifluoromethanesulfonyl)imide (LiTFSI) and battery grade propylene carbonate (PC) were purchased from Solvionic. Carbon-coated lithium iron phosphate (LiFePO_4_) was purchased from Aleees. Carbon black (C65) was purchased from Timcal.

### Synthesis of Single-Ion Polymeric Particles by Polymerization in Disperse Media

A 250 mL three-neck flask equipped with a reflux condenser, N_2_ inlet, temperature probe, and three feeding inlets was first charged with 70 g of Milli-Q water, pre-heated to 80 °C, and purged with N_2_ for 20 min. Then the feeding of monomers and redox initiators was started at the same time. Feedings were maintained for 3 h under continuous N_2_ purging, and then the system was allowed to react for an additional 1 h. Table 1 contains the amounts used for the polymerization. After polymerization, the polymeric nanoparticle dispersion was filtered with an 80 μm nylon mesh to remove coagulated nanoparticles. The amount of coagulum was calculated based on the total solid content of the dispersion and was less than 1 wt %. The dispersions were dialyzed against Milli-Q water using Spectra-Por 4 membranes (MW cut-off 12000–14000 Da) to remove unattached ionic species.

**Table 1:** Formulation of the Dispersion Polymerization Reaction

ID	initial charge [g]	feed 1 [g]	feed 2 [g]	feed 3 [g]	size [nm]	PDI
1	water [70]	LiMTFSI [2], ascorbic acid [0.228], water [10]	MMA [9.196], EGDMA [0.184]	TBHP [0.116], water [10]	200	0.079
2	water [70]	LiMTFSI [1], ascorbic acid [0.228], water [10]	MMA [9.196], EGDMA [0.184]	TBHP [0.116], water [10]	120	0.080
3	water [70]	LiMTFSI [0.5], ascorbic acid [0.228], water [10]	MMA [9.196], EGDMA [0.184]	TBHP [0.116], water [10]	95	0.075

### Titration of the LiMTFSI Surface Functionalities

A small volume of the dialyzed latexes were passed through a Dowex Marathon MSC cation exchange resin to exchange the lithium ions of the sulfonimide groups with titratable acidic protons. The concentration of acidic protons was measured using conductometric titration with a 5 mM NaOH aqueous solution. LiMTFSI incorporation was calculated as *n*/LIMTFSI_theor_, where *n* is number of moles of NaOH used in the titration until the end point and LIMTFSI_theor_ is the theoretical number of moles of LIMTFSI in the formulation.

### Preparation of the PEO/LiTFSI-Based Nanocomposite Electrolytes

A 5 wt % aqueous solution of poly(ethylene oxide) was mixed with a predetermined amount of LiTFSI (25% of PEO weight). Then the particle latex was mixed directly with the PEO/LiTFSI solution. The aqueous mixtures were casted onto silicon molds, and the water was removed by evaporation, producing self-standing composite polymer electrolyte films. The samples PEO/LiTFSI-based nanocomposite electrolytes were dried inside a Buchi high vacuum oven at 90 °C for 18 h before any further characterization.

### Preparation of the PC-Based Gel Nanocomposite Electrolytes

Freeze-drying was used to remove water from the dialyzed nanoparticle dispersion. The obtained fine white powder was further dried under reduced pressure at 90 °C for 18 h and transferred into an argon-filled glovebox. Then the particles were mechanically mixed with dry propylene carbonate (20 to 60 wt % of LiPNPs in PC) to obtain the gel nanocomposite electrolytes.

### Physical–Chemical Characterization

DMA experiments were performed on a PerkinElmer DMA 8000 in tension mode with a heating rate of 5 °C min^–1^, at 1 Hz frequency, at a strain of 25 μm, and in a N_2_ atmosphere. Small-amplitude oscillatory experiments were performed in a stress-controlled Anton Paar Physica MCR101 rheometer, and the experiments were carried out using 25 mm parallel plate geometry. All the experiments were conducted under linear viscoelastic conditions for the studied temperature range (strain = 0.5% and frequency 1 Hz). DSC experiments were performed on a Netzsch DSC 214 Polyma, using a heating rate of 5 °C min^–1^. Analysis was performed on second heating curves using Proteus software and AutoEvaluation. Broadband dielectric spectra in the frequency range of 10^–1^ to 10^6^ Hz were measured using an Novocontrol Concept-80 system, which includes an Alpha-A impedance analyzer and a Quatro Cryosystem temperature control unit. The samples were placed between stainless steel parallel plates with a 20 mm diameter, and the separation between the electrodes was determined by the film thickness, approximately 0.2 mm. The samples were placed inside the cryostat in a dry nitrogen atmosphere. The samples were equilibrated for at least 15 min after each temperature step to achieve thermal stabilization within 0.2 K. Electrochemical impedance spectra were measured, applying a 10 mV perturbation in a frequency range of 10^–1^ to 10^6^ Hz with an Autolab 302N potentiostat galvanostat, which includes a FRA32 M module and a Microcell HC temperature control unit. The samples were placed between gold-plated parallel plates with a 9 mm diameter, and the separation between the electrodes was determined by the film thickness, approximately 0.2 mm.

### Pulsed Field-Gradient Nuclear Magnetic Resonance

PFG-NMR experiments were performed on a Bruker Avance III wide-bore NMR spectrometer equipped with a 5 mm Diff50 probe. The maximum strength of the gradient amplifier was 29.4 T/m. ^19^F and ^7^Li diffusion coefficients were measured with separate RF coils which were tuned to the target nuclei frequency. The diffusion time was 100 ms for both ^1^H and ^19^F, and the gradient pulse durations were 10 ms and 5 ms for ^19^F and ^7^Li, respectively. A sample amount of approximately 70 mg was first packed into a 4 mm ZrO_2_ magic-angle spinning (MAS) rotor, and the packed rotor was then inserted into a 5 mm glass tube for diffusion measurement. A pulsed-field gradient stimulated echo (PFG-STE) sequence was used to obtain the diffusion coefficient.^35^ The sample temperature in the probe was calibrated in a temperature range of 0–70 °C, using the relative chemical shift separation between the OH and CH_3_ resonances of dry methanol.^36^

### Coin Cell Assembly

A composition of 60 wt % of carbon-coated LiFePO_4_, 30 wt % of PEO-NP10, and 10 wt % of carbon black was used for cathode preparation. First, powders of active material and carbon black were gently mixed in a hand mortar and successively added to a 5 wt % aqueous solution of PEO-NP10. The suspension was homogenized using an Ultraturrax mixer for 1 h. The aqueous slurry was cast onto a carbon-coated aluminum current collector using a doctor-blade with a blade height of 300 μm. The electrodes where dried in an oven at 50 °C. A PEO-NP10 film was hot-pressed onto the electrodes (70 °C at 10 bar for 10 min), dried at 70 °C/high vacuum for 24 h, and transferred to the glovebox. The areal capacity of the LFP cathode was 0.6 mAh cm^2^. LiFePO_4_/PEO-NP10/lithium metal coin cells were assembled in the glovebox. The coin cells were cycled at 70 °C at a constant C/10 current regime between 2.5 and 3.8 V versus Li^+^/Li. Lithium symmetrical coin cells were assembled inside the glovebox by sandwiching a PC-NP20 or a PC-NP30 film in between two lithium metal disks.

## 3 Results and Discussion

### Synthesis of Lithium Sulfonamide Functional Poly(methyl methacrylate) Nanoparticles

**Figure 1:**
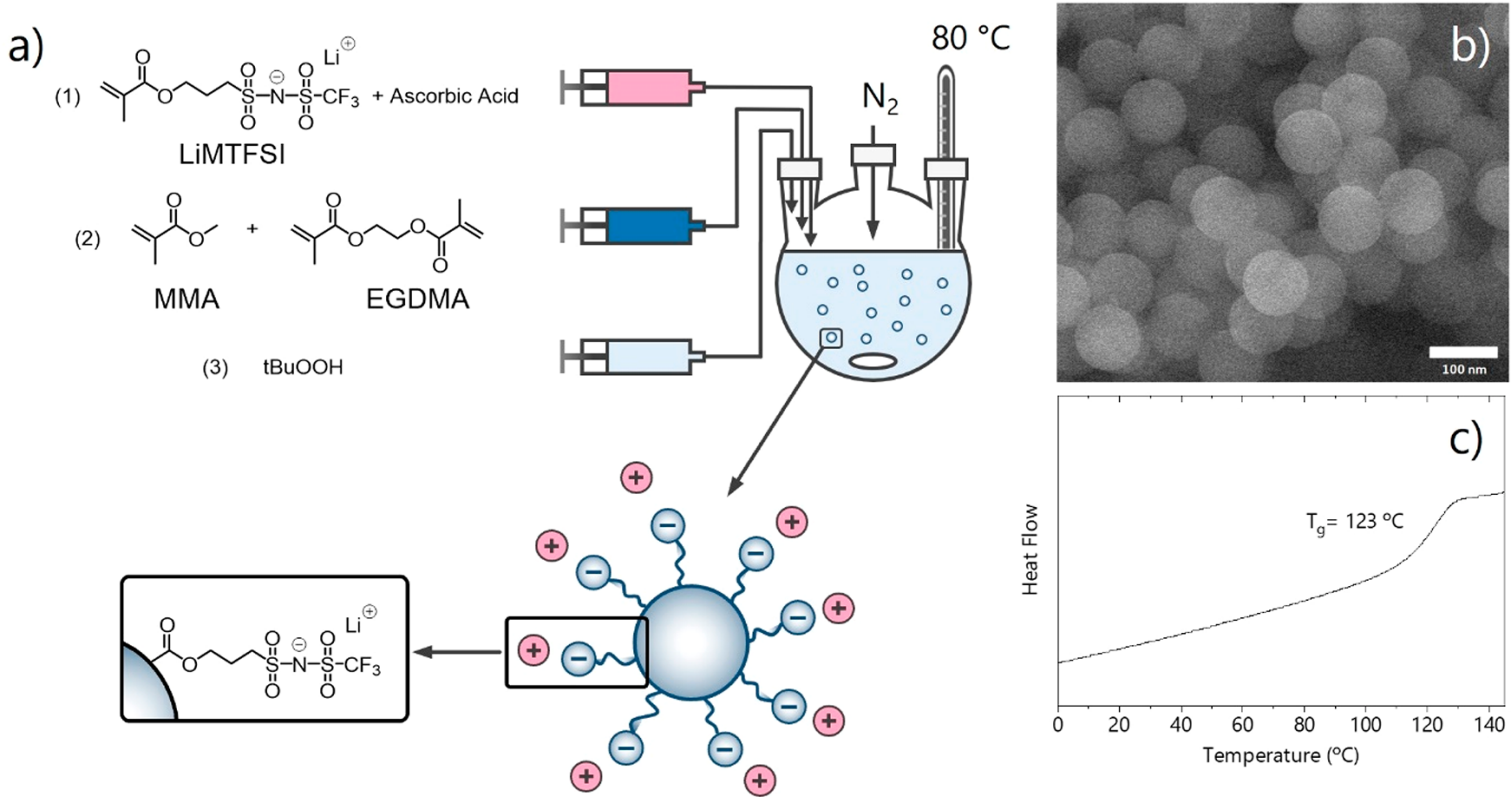
(a) Schematic representation of the polymeric nanoparticle synthesis and structure. (b) FESEM image of the polymeric nanoparticles. (c) DSC trace of the polymeric nanoparticles between 0 °C and 145 °C, 2nd heating at 5 °C/min.

As indicated previously, our goal here was to develop a one-pot synthetic method to obtain polymer nanoparticles which include lithium sulfonamide groups on their surface. These groups are the preferred option nowadays for the development of the so-called single-ion lithium conducting polymers due to the high charge delocalization of the sulfonamide anion which benefits lithium cation mobility. In our approach, single-ion nanoparticles were obtained in the form of a colloidal dispersion in water, commonly defined as latex, adapting a semibatch emulsion polymerization strategy reported in detail elsewhere.^19−21^ Briefly, the reactor was initially charged with water, purged with a gentle flow of N_2_, and heated to 80 °C. Three streams were injected to the reactor: the first contained the main monomer (methyl methacrylate, MMA) and the cross-linker (ethylene glycol dimethacrylate, EGDMA); the second contained the functional comonomer (LiMTFSI) and the reductant of the redox pair used to generate radicals (ascorbic acid); the third stream was the oxidant (*tert*-butyl hydroperoxide, TBHP) of the redox pair. The solids content of the synthesized latex was 5 wt %. Notably, surfactants were not used in the emulsion polymerization process, and the stability of the latex was achieved by the incorporation of the functional monomers (LiMTFSI) to the surface of the polymer particles and the electrostatic repulsion of the negatively charged particles as schematically shown in Figure 1. Figure 1(a) shows a schematic representation of the polymeric nanoparticle synthesis. The prepared latexes were purified by dialysis to remove unreacted monomers. The ionic monomer LiMTFSI served multiple functions of (1) providing colloidal stability to the dispersion of polymeric particles, (2) providing the desired functionality into the surface of the polymer particles, and (3) control of the polymer particle size by adjusting its concentration in the reaction formulation. Interestingly, we were able to control the size of the polymer nanoparticles by changing the LiMTFSI concentration in the reaction formulation. Dynamic light scattering (DLS) revealed a correlation between the LiMTFSI concentration in the reaction formulation and the number-average size of the particles. The size increased from 95 to 200 nm when the LiMTFSI concentration in the reaction formulation decreased from 2 g to 0.5 g. All samples showed a narrow polydispersity index (PDI), as shown in Table 1.

Field emission scanning electron microscopy (FESEM) images confirmed the particle size as measured by DLS and showed uniform spherical morphology of the polymeric particles; see Figure 1(b). Among the different particles available, we selected the one with smaller size for further study (*d* = 95 nm, PDI = 0.075). The particles were characterized by a single glass transition temperature (*T*_g_) at 122 °C, Figure 1(c). Besides the morphological characterization, a surface characterization of the particles was carried out to verify their functionality. By means of an acid–base titration method, we estimated that 80% mol of LiMTFSI monomer resides on the surface of the particles. Using this information, we calculated a surface density of five sulfonamide functional groups per nm^2^. The measured density value is higher than those achieved by Agrawal et al. via an inorganic nanoparticle functionalization process (1.3 chains per nm^2^),^28^ thus demonstrating that our simple one-step method can replicate the result of a more complex synthesis of functionalized inorganic nanoparticles. Overall, the semibatch emulsion polymerization in the disperse media process described here leads to lithium sulfonamide functional poly(methyl methacrylate) nanoparticles of particle sizes between 90 and 200 nm. These latexes could be lyophilized to obtain polymer nanoparticles in the form of powders. In the next sections, the potential use of these functional nanoparticles in composite electrolytes for lithium batteries will be investigated.

### Composite Solid All-Polymer Electrolytes Based on Poly(ethylene oxide) and Single-Ion Polymeric Nanoparticles

**Figure 2:**
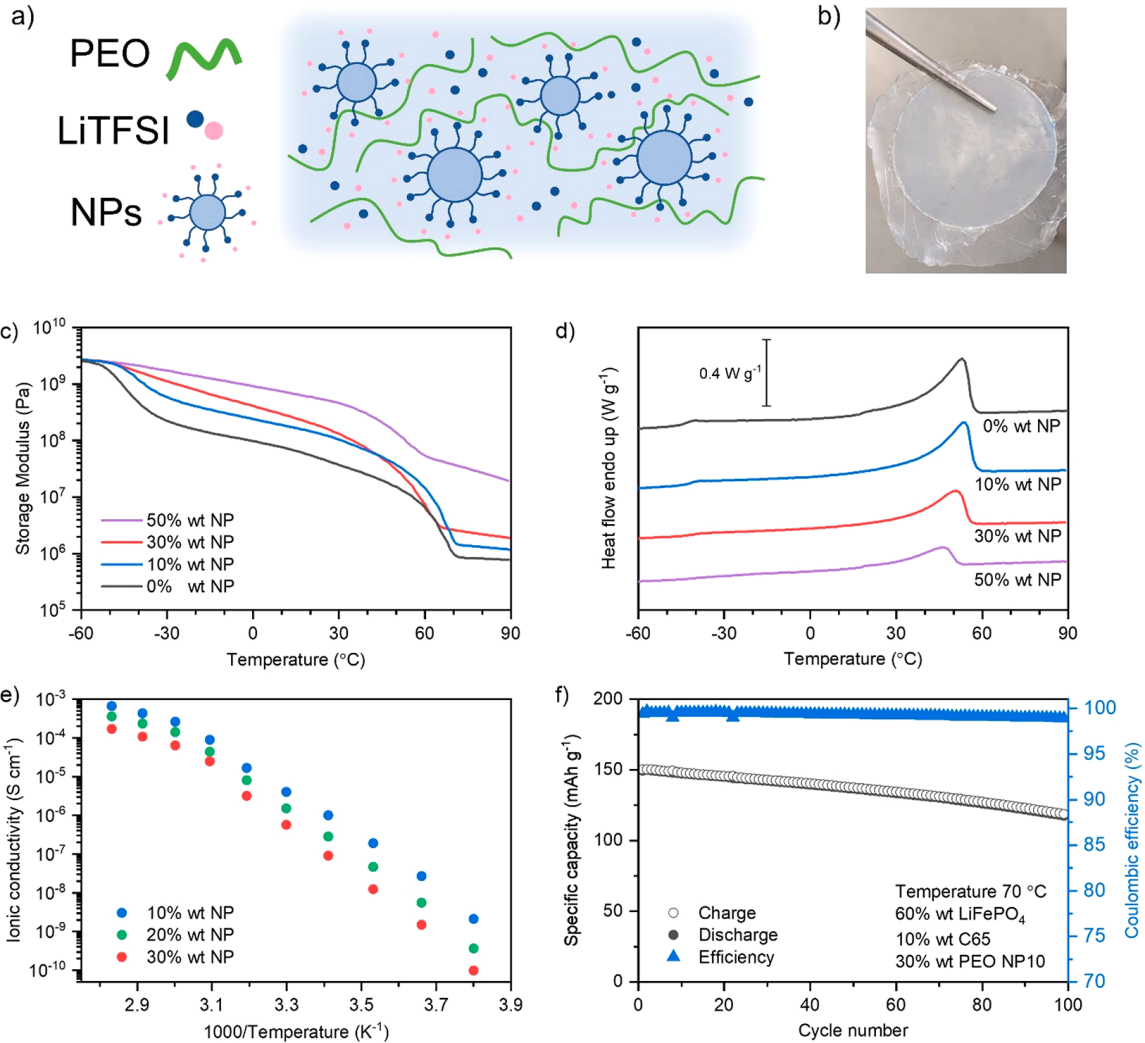
Solid polymer electrolytes based on poly(ethylene oxide): LiTFSI (75:25 wt %) and single-ion nanoparticles (NPS) with loadings from 0 to 50 wt %. (a) Schematic representation of the composite electrolyte. (b) Photography of a composite electrolyte. (c) Plot of the storage modulus (*E*′) as a function of temperature. (d) Plot of DSC showing glass transitions and melting peaks, 2nd heating at 5 °C min^–1^. (e) Plot of ionic conductivity (σ) as a function of temperature. (f) Plot of specific capacity versus cycle number of a Li/PEO-NP10/LiFePO_4_ cell at 70 °C, electrode active loading 0.6 mAh cm^2^.

Solid electrolytes based on poly(ethylene oxide) (PEO) are considered reference materials for solid-state cells, and it is speculated that commercial solid-state batteries from Bolloré employ PEO-based electrolytes.^29^ However, the ionic conductivity of PEO does not reach usable levels (10^–4^ S cm^–1^) until melting of the polymer semicrystalline regions (*T*_m_ ∼ 60 °C). At these high operating temperatures, PEO mechanical properties are lost and different strategies such as PEO cross-linking, blending, or the use of block copolymers have been proposed to circumvent this issue. On the other hand, the mechanical properties of PEO polymer electrolytes can be improved by preparing composites using nanosized reinforcement inorganic materials (SiO_2_ or TiO_2_ nanoparticles).^8,9^ In this work, the single-ion polymeric nanoparticles were investigated here to the same end as reinforcement fillers improving the mechanical properties of PEO polymer electrolytes. For this purpose, we took as a reference electrolyte, a high molecular weight PEO (average MW 900 000 g/mol) with 25 wt % LiTFSI with respect to the PEO weight. The concentration of single-ion nanoparticles in the composites was varied between 10 and 50 wt % with respect to the total weight. The samples were named as follows: PEO-NP10, PEO-NP20, PEO-NP30, and PEO-NP50 (the number represents the weight percent of polymer particles in the composite). The composite electrolytes were prepared easily by mixing the particle latex directly with an aqueous solution of lithium bis(trifluoromethanesufonyl)imide and poly(ethylene oxide), Figure 2(a). Interestingly, the polymer latexes did not lose colloidal stability upon mixing with the polymer/lithium salt solution. The aqueous mixtures were cast onto silicon molds, and the water was removed by evaporation, producing self-standing composite polymer electrolyte films. This preparation process is very convenient and green because it does not require the use of solvents other than water.

Dynamic mechanical analysis (DMA) was used to determine the impact of the nanoparticle content on the modulus of the PEO-based composite electrolytes. Figure 2(b) shows the plot of the storage modulus between −60 °C and 90 °C, consisting of multiple characteristic plateaus. The low temperature plateau below −50 °C corresponds to the glassy state and is equal for all samples to approximately 2.0 × 10^9^ Pa. This suggests that at low temperatures the stiffness of the samples is related to the PEO matrix and is independent of the nanoparticle concentration. As the samples are heated through the glass transition temperature of the PEO, between −50 °C and −40 °C, the stiffening effects of the nanoparticles that result from the PEO matrix–particle interactions become visible, seemingly reducing the storage modulus loss at the glass transition for higher particle concentrations. As the concentration increases, the softening associated with PEO transitioning to a rubbery state is effectively limited. At approximately 40 °C, all samples show a sharp loss in modulus corresponding to the PEO crystalline region melting, showing that the sample stiffness is related to both nanoparticle concentration and crystallized PEO. Above 70 °C, the nanoparticles alone still deliver a significant stiffening effect on the PEO matrix, increasing the storage modulus from the 10^6^ Pa range to 10^7^ Pa range. Figure 2(c) shows differential scanning calorimetry (DSC) curves of the PEO-based composite electrolytes. The graph shows that adding nanoparticles decreases the change in the heat flow at the glass transition around −45 °C, consistent with the DMA results. In addition, the melting peak of the PEO crystalline phase around 50 °C decreases as the concentration of nanoparticles increases. We note that while the transition signal and the melting peaks are weakening, the calculated values are relatively constant for all but the PEO-NP50 sample. The glass transition established from curve analysis is −42.0 ± 1.1 °C for PEO-NP0 through PEO-NP30, and −36.5 °C for PEO-NP50; see Table S1. Using a heat of fusion for 100% crystalline PEO of 196.6 J g^–1^,^30^ the percent crystallinity of the PEO regions was found to be approximately 45% for all but the PEO-NP50 sample, where it was closer to 35%. While not totally conclusive, it does suggest that the high concentration of particles in the PEO-NP50 sample limits the crystallization of PEO matrix compared to the other samples.

Next, the conductivity was estimated from broadband dielectric spectroscopy (BDS) using the DC plateau from spectra in the conductivity representation. Figure 2(d) shows the plot of ionic conductivity between −10 °C and 80 °C, for PEO-NP10, PEO-NP20, and PEO-NP30. PEO-NP0 and PEO-NP50 were excluded due to low modulus and low conductivity, respectively. The electrolyte with the lowest polymeric nanoparticle loading, PEO-NP10, showed the highest ionic conductivity, 1.0 × 10^–6^ S cm^–1^ at 20 °C and 6.6 × 10^–4^ S cm^–1^ at 80 °C. Increasing the nanoparticle loading decreased the ionic conductivity for all samples. The lowest ionic conductivity values were measured for sample PEO-NP30, 9.0 × 10^–8^ S cm^–1^ at 20 °C, and 1.7 × 10^–4^ S cm^–1^ at 80 °C. The conductivity reduction with increased nanoparticle wt % is correlated with an overall stiffening of the samples, an increased tortuosity of the lithium ions path due to the presence of the particles, and overall decrease of mobile ion concentration. Finally, the electrochemical performance of the polymer nanoparticle composites was evaluated in LiFePO_4_ lithium metal cells where PEO-NP10 was used both as electrolyte material and binder for the positive electrode. The LFP loading in the cathode was 0.6 mAh cm^2^. Figure 2(f) shows long-term cycling performance at a constant current rate of C/10. After 100 cycles, the capacity retention was found to exceed 83% of the initial capacity, and the Coulombic efficiency approached 100%, demonstrating excellent cell performance of the composite electrolytes.

### Composite Gel Electrolytes Based on Single-Ion Polymeric Nanoparticles and Propylene Carbonate

**Figure 3:**
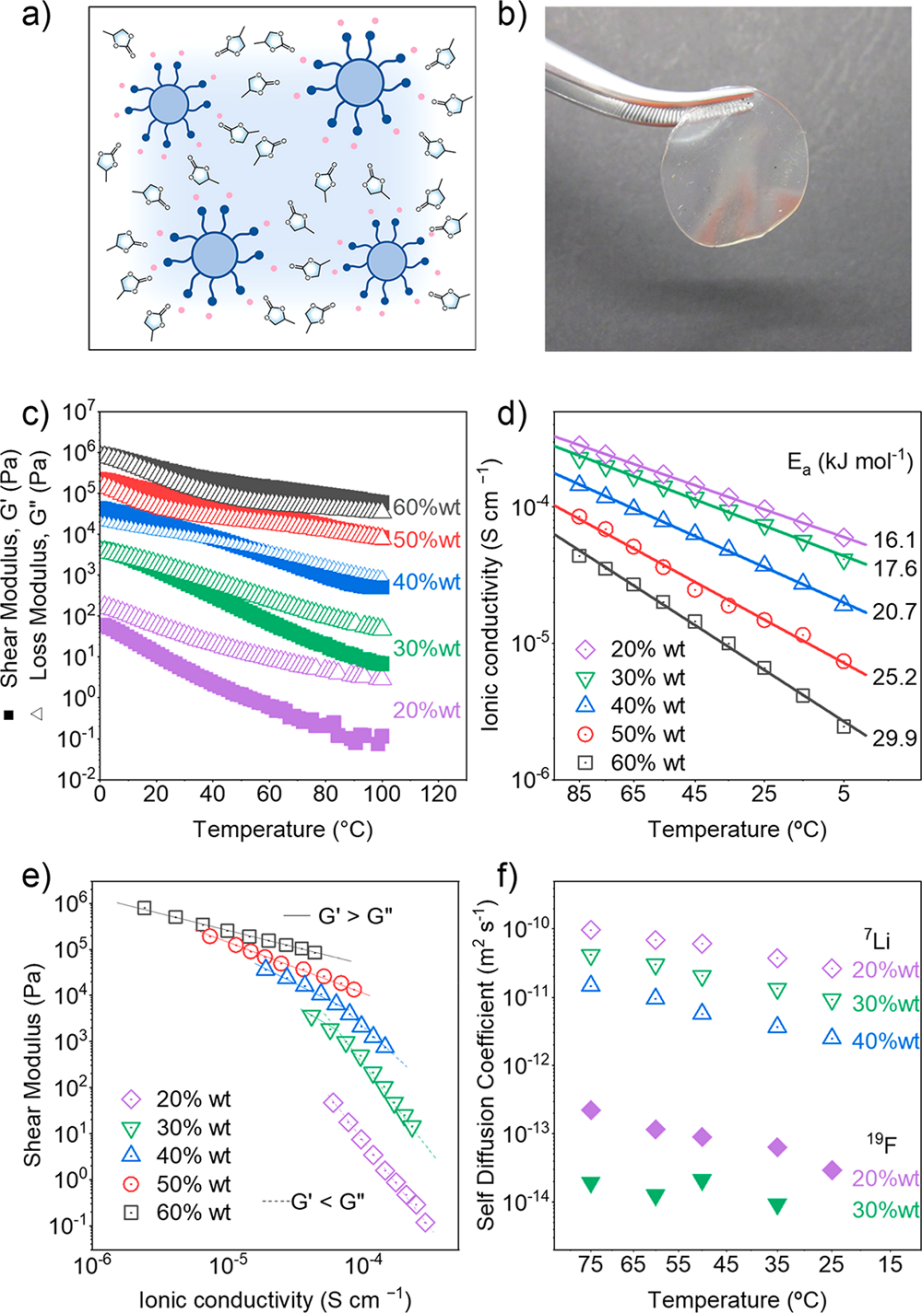
Gel electrolytes based on propylene carbonate and single-ion nanoparticles. Nanoparticle loadings are between 20 and 60 wt %. (a) Schematic representation of the composite electrolyte. (b) Photography of a composite electrolyte. (c) Plot of shear (*G*′) and loss (*G*′′) modulus as a function of temperature. (d) Plot of ionic conductivity (σ) as a function of temperature. (e) Plot of shear modulus (*G*′) as a function of the ionic conductivity (σ). (f) Plot of self-diffusion coefficients for ^7^Li and ^19^F as a function of temperature.

Gel polymer electrolytes are semisolid electrolytes which combine the mechanical and structural properties of polymers and the ionic conductivity of organic solvent or ionic liquid-based electrolytes. Gel polymer electrolytes can be prepared using a variety of materials as thickening agents such as polymer networks, supramolecular polymers, or block copolymers. Inorganic nanoparticles can be also used to obtain gel electrolyte. When gel electrolytes are combined with lithium single-ion conductors, a unique combination of high transference number and high ionic conductivity near room temperature can be achieved.^30^ In this work, our dry polymer particles were directly mixed with propylene carbonate (PC) in ratios between 20 and 60 wt % to obtain composite gel electrolytes. The nanoparticle/PC mixtures were coded as PC-NP20, PC-NP30, PC-NP40, PC-NP50, and PC-NP60 where the number represents the wt % of polymeric nanoparticles. Figure 3(a,b) shows a schematic representation of the composite electrolyte together with a picture of sample PC-NP60. At 20 wt % nanoparticle loading, a viscous liquid was produced, and adding more than 30 wt % caused gelation of the liquid electrolyte, while increasing the particle concentration further effectively solidified the electrolyte. Figure 3(c) shows the evolution of the shear moduli for the different samples as a function of temperature. Our results show that the shear storage modulus (*G*′) increased with the polymer particle loading of the composite electrolyte. At room temperature, the *G*′ of NP-20 was around 10 Pa while the value of *G*′ for NP-60 approached 10^6^ Pa, corresponding to a rubbery state. At higher temperatures, moduli of low loading samples (PC-NP20 and PC-NP30) decreased almost 3 orders of magnitude from initial values, while high loading samples decreased less than 1 order of magnitude over the same temperature range. Samples with the lowest particle loading showed shear loss modulus values (*G*′′) greater than *G*′ over the entire temperature range studied. PC-NP20 behaved as a free-flowing liquid throughout the experiment, suggesting that the particles are isolated in the PC matrix and cannot form percolating clusters to improve the mechanical properties of the composite. When increasing the particle loading to 30 wt %, the difference between *G*′′ and *G*′ decreases and NP-30 shows a crossing point just below room temperature. We hypothesize that the particles start to form percolating clusters at this stage, providing better mechanical properties to the electrolyte. For particle loadings equal to or higher than 40 wt %, *G*′ was higher than *G*′′, suggesting the formation of a mechanically robust network of polymeric particle clusters. With increasing temperature, the composite entered a free-flowing regime, as we observed a crossing point between *G*′ and *G*′′ slightly above 50 °C for NP-40. At this temperature, it is likely that the interparticle interactions of the suspected clusters are weakened, evidenced by the increased loss, disrupting their ability to stiffen the sample. Remarkably, PC-NP50 and PC-NP60 showed no crossing points between *G*′ and *G*′′ in the entire temperature range, suggesting that the polymeric particle network had percolated through the sample at these concentrations. The measured rheological properties are comparable with that of silica nanoparticle-reinforced PC electrolytes reported by Archer and co-workers.^28,31,32^ In the more ridged samples (*G*′ > *G*′′), there is a weaker dependence between modulus and temperature, i.e., *G*′ only decreases 1 order of magnitude between 0 °C and 100 °C. However, in the free-flowing samples (*G*′′ > *G*′), the dependence between the modulus and the temperature is significant, up to 3 orders of magnitude for PC-NP20.

After investigating the rheological properties of the nanoparticle/PC composites, we investigated its electrochemical properties. It is worth remembering that the ionic conductivity of our gels can be only due to the free lithium cations, therefore essentially making these gels single-ion conductors, because the anion is attached to the particles. We used sample resistance obtained from electrochemical impedance spectroscopy (EIS) to calculate the ionic conductivity of the composites, Figure 3(d) shows the plot of ionic conductivity at different polymer nanoparticle loadings in the temperature range of 5 °C to 85 °C. The electrolyte with the lowest polymeric nanoparticle loading, PC-NP20, shows the highest ionic conductivity, 9.5 × 10^–5^ S cm^–1^ at 25 °C and 2.8 × 10^–4^ S cm^–1^ at 85 °C. Increasing the nanoparticle loading decreased the ionic conductivity for all samples. The lowest ionic conductivity values were measured for sample PC-NP60 (6.6 × 10^–6^ S cm^–1^ at 25 °C and 4.4 × 10^–5^ S cm^–1^ at 85 °C). Conductivities could be easily fit using the Arrhenius equation. The activation energy increases with nanoparticle loading and is between 16.1 kJ mol^–1^ for PC-NP20 and 29.9 kJ mol^–1^ for PC-NP60. Figure 3(e) shows a direct comparison of the shear modulus with the ionic conductivity. Lines are drawn as a guide to the eyes; the top left point of each line corresponds to 5 °C whereas the bottom right point corresponds to 85 °C. The plot shows that the moduli of the nanocomposites extend over 7 orders of magnitude (10^6^ < *G*′ < 10^–2^ Pa). In contrast, ionic conductivity values extend over a smaller range of roughly 3 orders of magnitude (10^–6^ < σ < 10^–4^ S^1–^ cm). In the region of solidlike behavior (*G*′ > *G*′′), the conductivity is much more sensitive to temperature than the modulus. This suggests that the nanoparticle network is relatively stable, and that the heat energy is activating more ions for conductivity rather than softening the sample by reducing the nanoparticle–nanoparticle or nanoparticle–PC interactions. This is compared with the liquidlike behavior (*G*′′ > *G*′), where the opposite is taking place. Samples PC-NP30 and PC-NP40 show a transition between the two regimes, but at different temperatures, suggesting that there is some critical nanoparticle concentration for each temperature where heat will activate ions faster than disrupt nanoparticle interactions.

Next, the pulsed field-gradient NMR (PFG NMR) method was used to measure the self-diffusion coefficients of the lithium ions (*D*_Li_) and the TFSI moieties attached to the polymeric particles (D_F_). Because the only fluorine atoms present in the systems are contained in the TFSI moieties, we could directly associate the *D*_F_ to the self-diffusion of the anions (*D*_MTFSI_). We limited our investigation to three samples (PC-NP20, PC-NP30, and PC-NP40) because self-diffusion coefficients were too small to be measurable for higher NP concentrations. The ^7^Li NMR spectra showed a single signal, indicating the presence of a single coordination environment for the lithium ions in the samples. This evidence suggests that the lithium ions are dissociated from their highly delocalized MTFSI counter-ions and fully solvated by the propylene carbonate molecules. The temperature-dependent values of *D*_Li_ and *D*_MTFSI_ for the composite electrolytes are summarized in Table S2, and the Arrhenius plots are shown in Figure 3(f). Like the ionic conductivity values, the self-diffusion coefficients decreased with the particle loading for both *D*_Li_ and *D*_MTFSI_. The electrolyte with the lowest polymeric nanoparticle loading, PC-NP20, showed the highest *D*_Li_ of 9.75 × 10^–11^ m^2^ s^–1^ at 75 °C. Increasing the nanoparticle loading decreased the lithium self-diffusion for all samples. The lowest ionic diffusivity values were measured for sample NP-40, corresponding to 1.46 × 10^–11^ m^2^ s^–1^ at 75 °C. All samples showed *D*_MTFSI_ values that are 3 to 4 orders of magnitude smaller than the *D*_Li_. *D*_MTFSI_ for PC-NP20 was 2.24 × 10^–13^ m^2^ s^–1^ at 75 °C. When increasing the particle loading, the *D*_MTFSI_ value at room temperature was too small to be measurable. Interestingly, the measured lithium self-diffusion coefficients were only slightly smaller than the values reported by Hayamizu et al.^33^ for a 1 M LiTFSI in PC. However, the *D*_MTFSI_ corresponding to our anionic nanoparticles were several orders of magnitude lower than the *D*_TFSI_ values reported by the same authors for 1 M LiTFSI in PC. This is due to well established dynamics of anions in traditional dual ion solutions, where anions are usually faster than that of lithium ions that travel with a large solvation shell. However, the ionic mobility in our system is inverted, with Li being the more mobile of the two ions, proving that tethering the anions to a particle surface is an effective strategy for decreasing their motion.

**Figure 4:**
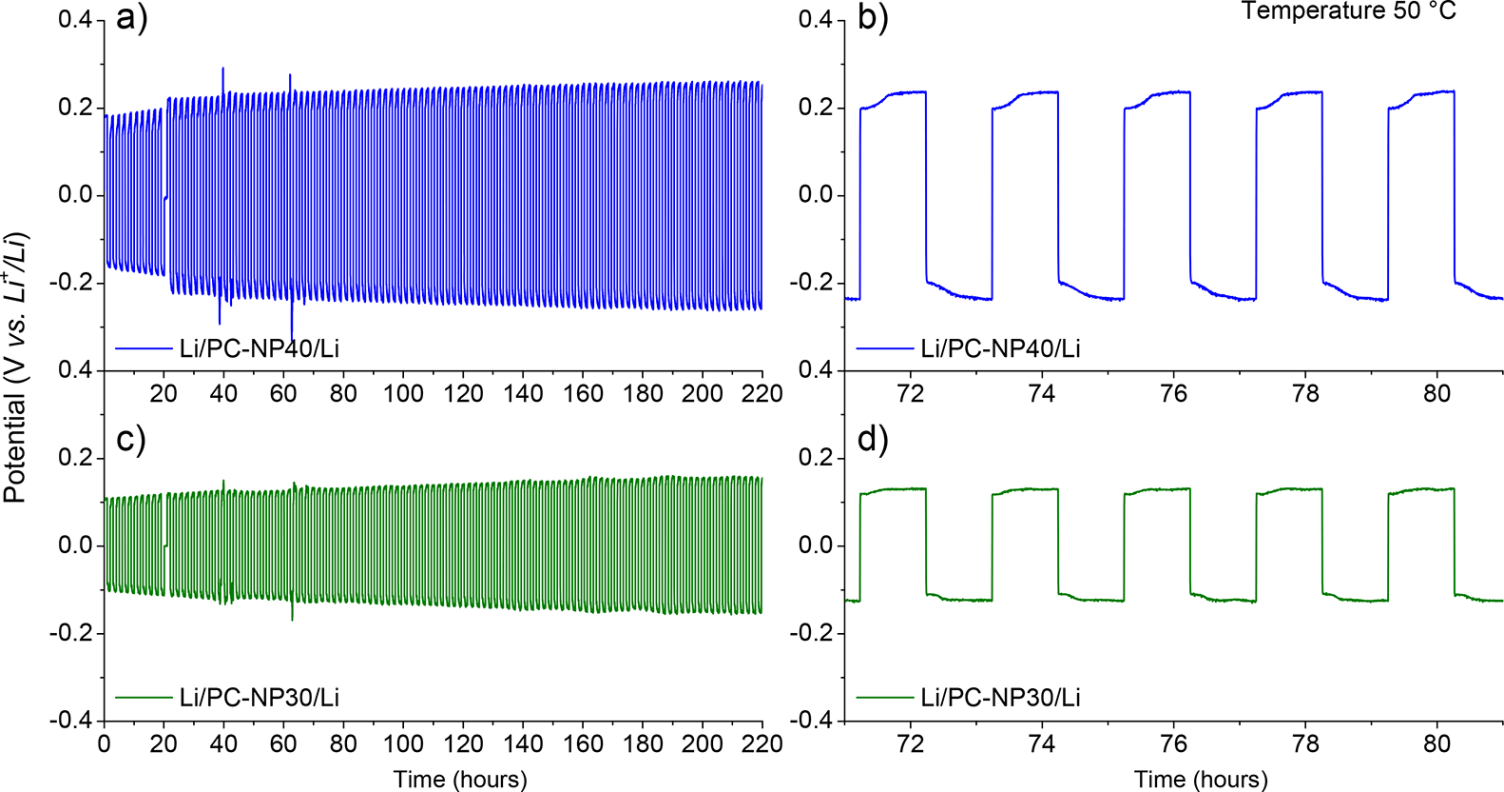
Gel electrolytes based on propylene carbonate and single-ion nanoparticles. Plot of the overpotential as a function of time for a symmetrical lithium cell cycled at 0.1 mA cm^–2^ at 50 °C with (a, b) PC-NP30 and (c, d) PC-NP40 electrolyte.

To confirm this, we measured the lithium transference number of PC-NP20 using the Vincent and Bruce method.^34^ The calculated value was *t*+ = 0.8 at 25 °C (Figure S1). This quick estimation of the lithium transference number suggests that our electrolyte does in fact behave very close to a single-ion conductor, bearing in mind that some small contribution may come from the migration of the anion-functionalized NPs.^15,16^ Overall, this shows the validity of our goal to obtain single-ion gel polymer electrolytes simply by blending the lithium sulfonamide PMMA functional polymer nanoparticles with an organic solvent such as propylene carbonate. To demonstrate the suitability of these composite electrolytes for lithium metal batteries, coin cells were assembled and cycled galvanostatically at 50 °C. Figure 4 shows the performance of PC-NP30 and PC-NP40 in a symmetrical lithium cell cycled at 0.1 mA cm^–2^. The recorded overvoltage was 100 mV and 125 mV for NP30 and PC-NP40, respectively. The test was carried out over a period of 220 h and showed a relative stable overvoltage with no short-circuits. Although these initial results are encouraging, further optimization work is required to achieve lower overvoltages at higher current densities comparable to commercial grade electrodes for lithium batteries.

## 4 Conclusions

In this work, we reported the synthesis of lithium sulfonamide surface-functionalized polymer nanoparticles by a semibatch emulsion polymerization process that can be scaled-up easily. The lithium sulfonamide functionalization was introduced into cross-linked poly(methyl methacrylate) by including into the typical emulsion polymerization formulation just the LiMTFSI comonomer. Polymer nanoparticles of sizes ranging from 90 to 200 nm were obtained in gram scale with a surface functionalization of five functional groups per nm^2^ in the form of stable polymer latex or dry powder of nanoparticles after lyophilization. The polymer nanoparticles were used to prepare two different composite solid electrolytes for lithium batteries. In the first example, we used the particle as nanoreinforcement, or filler, to improve the mechanical properties of a reference electrolyte based on high molecular weight PEO and 25 wt % LiTFSI. The particles delivered a significant stiffening effect on the PEO matrix (*E*′ > 10^6^ Pa at 80 °C) while the membranes retained high ionic conductivity values (σ = 6.6 × 10^–4^ S cm^–1^). In the second example, the particles were mixed directly with propylene carbonate without any additional salt. The obtained gel electrolytes were mechanically robust (up to *G*′ = 10^6^ Pa) and exhibited a lithium transference number close to unity. This case shows that the polymer nanoparticle can be used as a functional filler to obtain single-ion lithium conducting composite solid electrolytes. Finally, the two families of particle-reinforced electrolytes were tested in of symmetrical lithium cells and LiFePO_4_ lithium metal cells, showing promising performance. Balancing mechanical properties and lithium-ion mobility of an electrolyte remains an elusive target that will require further research efforts; however, in this work we present a promising new building block material to address this technological challenge.

## Supporting Information Available

DSC curve analysis, fitting results from Netzsch, self-diffusion coefficients table, and polarization and impedance spectra (PDF)
