# Supplementary material for: Single-Ion Conducting Polymer Nanoparticles as Functional Fillers for Solid Electrolytes in Lithium Metal Batteries
Source: ACS Appl Mater Interfaces. 2021 Nov 3;13(45):54354–62. doi: 10.1021/acsami.1c15771 (PMC8603348; doi:10.1021/acsami.1c15771)
Supplement: Supplementary file 1 — am1c15771_si_001.pdf [file am1c15771_si_001.pdf]

Supporting Information

Single–Ion Conducting Polymer Nanoparticles as Functional Fillers for Solid Electrolytes in Lithium Metal Batteries

Luca Porcarelli,[ab] Preston Sutton,[b] Vera Bocharova,[c] Robert H. Aguirresarobe,[a] Haijin Zhu,[b] Nicolas Goujon,[ab] Jose R. Leiza,[a] Alexei Sokolov,[cd] Maria Forsyth*[abe] and David Mecerreyes*[abe]*

* Corresponding authors

[a] POLYMAT, University of the Basque Country UPV/EHU, Joxe Mari Korta Center 20018, Donostia–San Sebastian, Spain

Email: luca.porcarelli@ehu.es

Email: david.mecerreyes@ehu.es

[b] ARC Centre of Excellence for Electromaterials Science and Institute for Frontier Materials, Deakin University, Melbourne, Australia

Email: maria.forsyth@deakin.edu.au

[c] Chemical Sciences Division, Oak Ridge National Laboratory, Oak Ridge, Tennessee 37831, United States

[d] Department of Chemistry, University of Tennessee, Knoxville, Tennessee 37996, United States

[e] Ikerbasque, Basque Foundation for Science, Maria Diaz de Haro 3, E–48011 Bilbao, Spain

Table S1 DSC curve analysis, fitting results from Netzsch

Sample	mass [mg]	PEO mass [mg]	Peak melt [°C]	T_g [°C]	Peak area [J/g]	X_c, PEO [%]
PEO-NPS50	7.1	2.663	46.5	-36.5	25.19	34.2
PEO-NPS30	8.6	4.515	51.0	-41.0	47.01	45.5
PEO-NPS10	7.8	5.265	53.6	-42.0	63.14	47.6
PEO-NPS0	5.8	4.35	53.1	-43.1	65.66	44.5

Table S2 Self–diffusion coefficients of the lithium–ions, the tethered anions, and lithium transference numbers for the nanocomposite electrolytes based on polymeric nanoparticle, nanoparticle loading between 20 and 40 wt%.

Temp	Sample		
(°C)	**NP–20**	**NP–30**	**NP–40**
	^{7}Li Self–Diffusion Coefficient (m^2 s^{-1})		
75	9.75×10^{-11}	4.18×10^{-11}	1.46×10^{-11}
60	6.86×10^{-11}	3.06×10^{-11}	9.57×10^{-12}
50	6.08×10^{-11}	2.07×10^{-11}	5.72×10^{-12}
35	3.75×10^{-11}	1.34×10^{-11}	3.64×10^{-12}
25	2.64×10^{-11}	9.35×10^{-11}	2.49×10^{-12}
	^{19}F Self–Diffusion Coefficient (m^2 s^{-1})		
75	2.24×10^{-13}	1.95×10^{-14}	–
60	1.16×10^{-13}	1.26×10^{-14}	–
50	8.91×10^{-14}	2.12×10^{-14}	–
35	6.39×10^{-14}	9.25×10^{-15}	–
25	2.93×10^{-14}	–	–
	Apparent Lithium transference number		
75	0.999	0.999	–
60	0.999	0.999	–
50	0.998	0.998	–
35	0.999	0.999	–
25	0.999	–	–

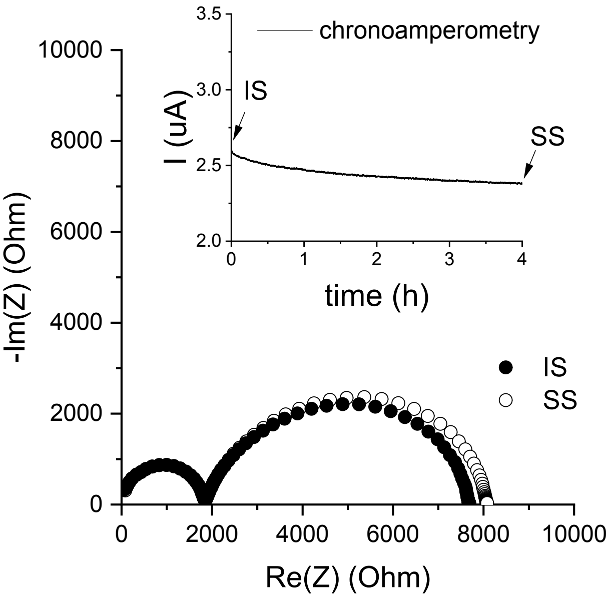

Figure S1 Polarization curve and impedance spectra collected to calculate the transference number with the Bruce-Vincent formalism
